# Lethal means safety for Asian American, Native Hawaiian, and Pacific Islander Veterans: Insights from key informants

**DOI:** 10.1371/journal.pone.0340962

**Published:** 2026-01-30

**Authors:** Lindsey L. Monteith, Evan R. Polzer, Ryan Holliday, Darrin M. Aase, Joseph A. Simonetti

**Affiliations:** 1 VA Rocky Mountain Mental Illness Research, Education and Clinical Center for Suicide Prevention, Aurora, Colorado, United States of America; 2 Spark M. Matsunaga VA Medical Center, VA Pacific Islands Healthcare System, Honolulu, Hawaii, United States of America; 3 Department of Psychiatry, University of Colorado Anschutz Medical Campus, Aurora, Colorado, United States of America; 4 Department of Physical Medicine and Rehabilitation, University of Colorado Anschutz Medical Campus, Aurora, Colorado, United States of America; 5 Firearm Injury Prevention Initiative, University of Colorado Anschutz Medical Campus, Aurora, Colorado, United States of America; Uniformed Services University of the Health Sciences, UNITED STATES OF AMERICA

## Abstract

**Aims:**

Asian American, Native Hawaiian, and Pacific Islander (AANHPI) Veterans are more likely to use suffocation and less likely use firearms in suicide deaths, relative to Veterans overall. Information to inform tailoring of lethal means safety (LMS) interventions for AANHPI Veterans is limited. We elicited subject matter experts’ perspectives regarding LMS among AANHPI Veterans, including awareness of differences in suicide methods and insights regarding LMS considerations.

**Method:**

Qualitative interviews were conducted with forty-three key informants in the Continental U.S. (CONUS), Hawaii, and Guam in 2022–2024. Interview transcripts were examined via deductive content analysis for this secondary analysis.

**Results:**

Key informants noted an increased use of hanging among AANHPI Veterans and in specific regions (e.g., Pacific Islands), though mention of hanging/suffocation was specific to key informants in Hawaii and Guam. Key informants noted that LMS efforts do not address hanging, emphasizing the need for resources that address hanging and that are tailored to local contexts. Some key informants also noted the lower use of firearms as a suicide method among AANHPI Veterans, sharing potential explanations for this, while expressing concerns that recent increases in firearm access in this population could lead to increases in firearm suicide deaths. Accordingly, key informants expressed a need to understand how cultural considerations could be incorporated into firearm LMS efforts for AANHPI Veterans.

**Discussion:**

Some subject matter experts, particularly those in the U.S. Pacific Islands, have awareness of the differences in suicide methods among AANHPI Veterans; however, increasing CONUS practitioners’ awareness of the increased use of hanging/suffocation as a suicide method among AANHPI Veterans may be warranted. Developing strategies to prevent suicide by hanging is crucial to preventing suicide among AANHPI Veterans. Considering potentially increased rates of firearm access among AANHPI Veterans, development of culturally responsive firearm suicide prevention efforts for this population is also warranted.

## Introduction

The suicide rate among Asian American, Native Hawaiian, and Pacific Islander (AANHPI) Veterans has increased over time [1,2]. A recent analysis also found that, among U.S. AANHPI adults, a history of military service was associated with elevated odds of reporting lifetime suicide attempts [3]. Considering these findings, alongside a prior review that delineated the need for additional knowledge regarding suicide risk and prevention among AANHPI Veterans [4], a multitude of studies have recently sought to understand drivers of suicide risk, contextual factors, and considerations for suicide prevention among AANHPI Veterans (e.g., [5]).

One important finding that has recently emerged from such research is stark differences in the most common methods of suicide death among AANHPI Veterans [6]. Specifically, firearm injuries account for a lower percentage of suicide deaths among AANHPI Veterans, compared to among Veterans overall (40.8% vs. 69.2%, respectively, in 2019; [6]). Conversely, suffocation accounts for a higher percentage of suicide deaths among AANHPI Veterans, relative to among Veterans overall (38.5% and 16.9%, respectively, in 2019; [6]). The extent to which suffocation is used as a suicide method is further elevated among specific subsets of the AANHPI Veteran population; for example, among females, suffocation accounted for a higher percentage of suicide deaths from 2015–2019 than firearm injuries [6]). Use of suffocation as a suicide method is also elevated among AANHPI Veterans residing in specific regions, such as the Northeastern U.S. [7]. Further concerning, in the U.S. Pacific Island Territory of Guam, where the majority of the populace identifies as Pacific Islander [8], hanging accounted for 82% of suicide deaths from 2012–2022 [9].

The vast majority of suffocation suicide deaths among AANHPI Veterans appear to be through hanging [10]. The specific ligature types used by AANHPI Veterans in hanging suicide deaths vary extensively, with most appearing to be those that are readily available (e.g., ropes, electronic cords, garments; [10]). The ubiquity of these methods in most households and in daily life underscores a challenge to preventing suicide through such methods, as approaches to prevent suicide through specific methods typically rely upon decreasing individuals’ access to such methods (i.e., lethal means safety [LMS]), which is considered an integral component of suicide prevention [11,12]. However, guidance specific to preventing hanging remains limited [12,13].

## Aims

While numerous studies have focused on suicide risk and prevention among AANHPI populations [14–16], limited work has focused on LMS considerations for AANHPI Veterans, with respect to hanging or other suicide methods. To accelerate knowledge regarding this understudied, yet clinically important domain, we sought to explore subject matter experts’ perspectives regarding lethal means and LMS among AANHPI Veterans, including their awareness of differences in suicide methods, as well as insights regarding LMS considerations for this population of Veterans.

## Methods

### Eligibility, recruitment, and screening

Data for this secondary analysis were analyzed as part of a broader study focused on suicide prevention among AANHPI Veterans in different U.S. regions. Key informants based in the Continental United States (CONUS; [5]) were recruited, along with key informants in Hawaii and Guam, given the large populations of AANHPI Veterans in these regions [17–19]. The target sample comprised individuals with expertise and/or experience relevant to suicide prevention with AANHPI Veterans. Specific inclusion criteria entailed having expert knowledge of AANHPI populations and/or experience working with AANHPI Veterans in CONUS, Hawaii, Guam, the Northern Mariana Islands, or American Samoa. Examples included providing suicide prevention services to AANHPI Veterans or AANHPI populations, conducting relevant research (e.g., published research on AANHPI Veterans or on suicide prevention in AANHPI populations), and other relevant experience (e.g., leadership in community healthcare organizations that provide services to AANHPI Veterans; involvement in public health-oriented suicide prevention initiatives in regions with large numbers of AANHPI Veterans). Exclusion criteria encompassed being unable to provide informed consent, per the judgment of the research team.

Key informants were recruited from September 12, 2022 to September 18, 2024. Potential key informants were initially identified through literature reviews and online searches and invited to participate by email. Professional listserv postings, social media, and snowball sampling were also used for recruitment. Regarding snowball sampling, at the end of each interview, key informants were asked if they knew anyone additional with relevant subject matter expertise (e.g., experience working with AANHPI Veterans in suicide prevention or an adjacent role) whom they would recommend for an interview. The research team contacted individuals identified by key informants as having relevant subject matter expertise to invite their participation, pending confirmation of their eligibility to participate. Individuals who were recruited to participate via snowball sampling, professional listserv postings, or social media were screened for eligibility by study staff, using a brief screening form developed for this study. During screening, study staff inquired about their experience working with AANHPI Veterans and AANHPI populations; having relevant experience (e.g., work experience or publication history) was required to participate.

### Procedures

Ethics approval to conduct this study was obtained from the Colorado Multiple Institutional Review Board and the VA Eastern Colorado Healthcare System Research and Development Committee (Protocol 20–4023). The IRB approved a waiver of documentation of consent for this study. Individuals who were eligible to participate were scheduled for a 1:1 appointment that occurred virtually, by phone, or in-person. Appointments began with informed consent, in which participants verbally conveyed informed consent to research staff. Informed consent was obtained from all participants prior to initiating study procedures.

Thereafter, an experienced qualitative interviewer used a semi-structured interview guide (S1) to conduct the qualitative interview [5]. The interview guide began by exploring the key informant’s reasons for participating, their current employment, and their experiences working with AANHPI individuals, including AANHPI Veterans. The guide covered a broad range of topics relevant to suicide prevention among AANHPI Veterans, such as cultural norms relevant to understanding suicide risk and prevention among AANHPI Veterans; the key informant’s perspectives on how well current suicide prevention initiatives incorporate such norms; and current efforts to prevent suicide among AANHPI Veterans, including helpful and unhelpful aspects, as well as how such initiatives could be adapted to reflect AANHPI Veterans’ needs, values, and cultures; suicide prevention considerations pertinent to AANHPI Veterans (e.g., where interventions should be delivered, by whom, and barriers and facilitators to implementation). The interview guide additionally explored geographical considerations for preventing suicide among AANHPI Veterans in the key informant’s region, considerations for specific interventions (e.g., Safety Planning [20]) with AANHPI Veterans (asked among respondents who were healthcare providers), and the most critical research needed to prevent suicide among AANHPI Veterans. Thus, although the interview guide did not include specific questions about LMS, there were opportunities for content related to LMS to emerge. The specific questions that most commonly elicited responses relevant to the present aims pertained to questions about risk and protective factors for suicide among AANHPI Veterans, the extent to which current suicide prevention efforts address the needs of AANHPI Veterans, Safety Planning considerations (e.g., experiences, challenges, and considerations), geographical considerations, research needed to prevent suicide among AANHPI Veterans, and questions regarding cultural norms and considerations.

Interviews were audio-recorded, professionally transcribed, and de-identified. Participants were compensated $50 for participating, except for those participating as VA employees or who declined compensation.

### Participants

Forty-three individuals consented and participated as key informants between September 21, 2022 and October 17, 2024. Key informants were associated with three geographic regions: CONUS (*n* = 14; 32.6%), Hawaii (*n* = 15; 34.9%), and Guam (*n* = 14; 32.6%). The sample included individuals who worked in VA (*n* = 18; 41.9%) and non-VA (*n* = 25; 58.1%) settings. Key informants’ roles varied, with over half (*n* = 23; 53.5%) providing direct clinical care (i.e., mental health care, suicide prevention services) currently or previously. For key informants who had not directly provided mental health care or suicide prevention services, many had been involved in community-based suicide prevention (e.g., Governor’s Challenges to Prevent Suicide among Service Members, Veterans, and their Families [21,22]) and/or had worked with AANHPI Veterans in other roles (e.g., transition assistance counselor, military chaplain, homeless services, social services, peer support, Veterans Service Organization leader, traditional healing) in the VA, Department of Defense, or the community. Approximately forty percent (*n* = 18; 41.9%) of the overall sample self-identified as having served in the U.S. military. While disclosure of U.S. military service was relatively infrequent among key informants who had provided mental health care or suicide prevention services (*n* = 3; 13.0%), the vast majority of participants who had not directly provided mental health care or suicide prevention services disclosed a history of military service (*n* = 15; 75.0%).

### Analysis

Three individuals (LLM, ERP, RH) analyzed interview transcripts and excerpts. Each had prior expertise conducting qualitative research on Veteran suicide prevention, including among AANHPI Veterans. Two of the analysts are licensed psychologists and doctoral-level researchers. The third analyst is a masters-level researcher with a background in medical anthropology who has contributed to a multitude of Veteran suicide prevention research studies. One analyst identified as AANHPI; none identified as Veterans.

Interview transcripts were analyzed via deductively driven content analysis [23,24]. Search terms used to review transcripts for content relevant to LMS were derived *a priori* through a concept-driven coding process [25], with terms drawn from pre-existing theory, research, and concepts related to LMS. Once the list of *a priori* search terms was developed and agreed upon, the analysts each reviewed a different subset of transcripts (*n =* 3 each; 9 total) to validate the completeness of the list and determine if any additional *post hoc* search terms (i.e., additional relevant terms that emerged inductively in reading transcripts) should be added. Three search terms were added through this process. The updated list was systematically applied to each transcript and encompassed the following search terms: firearm, gun, hang*, harm*, lethal, ligature, means, medication, method, overdos*, pill, poison*, suff*, weapon. Analysts then began by individually and systematically searching transcripts for all usages of each search term. When the search term was found in a transcript, the analyst noted the direct quotation and the context (i.e., the question that elicited the content), as well as the professional role and geographic region of the key informant. This information was captured in a Microsoft Excel spreadsheet, where these details were collated for further analyses. Given the dichotomous, manifest-level coding of this analysis (i.e., whether the search term was present or not), intercoder reliability was not calculated. Each analyst independently reviewed all compiled excerpts and contextual information, independently formed their impressions, and met to deliberate and discuss findings, interpret the results, and reach consensus regarding findings.

## Results

### Content analysis

Terms related to LMS were identified in 39.5% of transcripts (*n* = 17), including 8 CONUS transcripts, 6 Hawaii transcripts, and 3 Guam transcripts (Table 1). Of the transcripts in which search terms were identified, the vast majority were from key informants who had directly provided mental health care or suicide prevention services (*n* = 11; 64.7%); approximately one-third of the remaining transcripts (*n* = 6; 35.3%) were from key informants who had not previously worked in clinical roles involving the direct provision of mental health care or suicide prevention services.

**Table 1:** Percentage of Key Informant Transcripts with Coded Content, by Region.

Region	*n*	%
Continental United States (*n* = 14)	8	57.1
Hawaii (*n* = 15)	6	40.0
Guam (*n* = 14)	3	21.4

Regarding terms identified (Table 2), “gun” was the most frequent term coded (identified in 27.9% of transcripts), followed by “means” (16.3%), and “firearm” and “hang*” (each identified in 11.6% of transcripts). A full list of code frequencies and examples is in Table 2.

**Table 2:** Search Terms, Frequencies, and Examples.

Term	n	%	Example of Coded Content
*Gun*	12	27.9%	“Most of them will accept *gun* locks from me. And other ones will say that they will give lethal means to another family member.”
*Means*	7	16.3%	“So usually in my initial sessions with most people, I will talk about that, the difference between ideation and actually like having a plan with *means*.”
*Firearm*	5	11.6%	“…everyone’s journey is so different, but the journey ends the same. And the journey is expedited by these two things, drugs, or three things; drugs, to include alcohol, and *firearms*.”
Hang*	5	11.6%	“But it’s still, if I were a betting man, it would be much more geared to *hanging*.”
*Medication*	3	7.0%	“The stark difference is, a lot of White-identifying older adults turn to their spouses, their partners, to—for their Safety Plan, both in terms of distraction, who would they turn to in need, someone to manage their *medications*, does all of these things. However, for Asian-American individuals, that perceived burdensomeness, or that closeness, sometimes interferes with them—Asian-American individuals or Asian individuals putting the people closest to them on that Safety Plan.”
*Lethal*	3	7.0%	“We do have access to *lethal* means in [state] and [military base] in [state], which we see a higher rate of suicide, or higher number of suicides in those states.”
*Weapon*	3	7.0%	“[State] you can purchase a *weapon*, but it’s really—this is one of the toughest states when it comes to gun laws.”
Overdos*	2	4.7%	“Here in [State], in Asia, it’s usually hanging or *overdose*.”
*Pill*	2	4.7%	“Instead of talking specifically about diagnosis, talking about coping and how to cope with just life in general because life is stressful. And stressful things happen in people’s lives every day. So that they know that it’s not just suicide, right? Or I’m gonna take all these *pills* and go to sleep and never have to worry about things again.”
*Method*	2	4.7%	“Now of course it’s a mental health problem, but it’s also a problem – suicide is a violent activity. And it’s something that a lot of mental health professionals don’t quite grasp that public health professionals do. And because it’s a violent activity, we gotta pay attention to the means that people take, the *method* of suicide.”
*Ligature*	1	2.3%	“And then specifying to like *ligatures*, issues with hanging, I think that would be great research.”
Suff*	1	2.3%	“Hanging is, *suffocation* is the top, it’s the number one.”
Poison*	0	0.0%	N/A
Harm*	0	0.0%	N/A

Among 17 key informants for whom at least one search term was identified, most used multiple search terms in their interview (median = 2; mean = 2.7; SD = 1.8; range = 1, 6). Plural versions of these search terms were also identified through the terms included here. All search terms were identified *a priori*, except for *weapon*, *gun*, and *pill*.

* Truncated search term.

**Fig 1:**
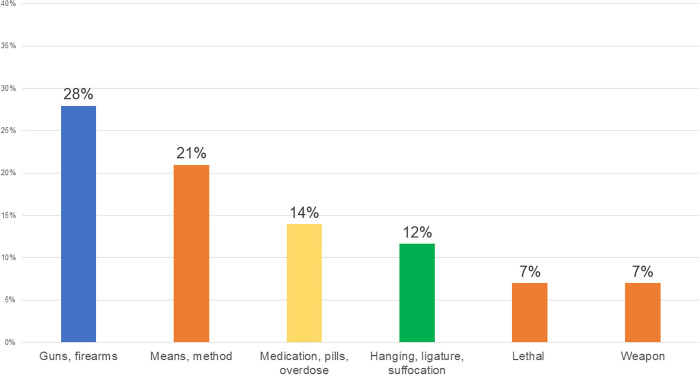
Percentage of Key Informant Transcripts with Each Search Term Category. Orange bars refer to non-specific methods.

Search terms were collapsed into conceptually-driven categories (specific method categories were grouped to reflect those reported in the annual VA Suicide Prevention Report [1]). The most common method category was gun/firearm (27.9% of transcripts; *n* = 12), followed by medication, pill, overdose (14.0%; *n* = 6), then hang*, ligature, suffocation (11.6%; *n* = 5; Fig 1). More general term categories were also noted, with means/method (20.9%; *n* = 9) more common than lethal (7.0%; *n* = 3) or weapon (7.0%; *n* = 3).

Interestingly, mentions of certain terms varied by region. Specifically, hanging, ligature, or suffocation were discussed by key informants in Hawaii (*n* = 2) and Guam (*n* = 3); however, no key informants in CONUS mentioned these. In contrast, firearms were discussed by key informants across all three geographic regions (CONUS: *n* = 6; Hawaii: *n* = 4; Guam: *n* = 2), particularly those in CONUS.

#### “*Less guns, other methods usually come into play, and hanging has been the predominant one of those*”: Differences in suicide methods.

Key informants discussed differences in suicide methods, regionally between CONUS and the U.S. Pacific Islands, and between AANHPI and non-AANHPI populations. In particular, they noted the increased use of hanging and lower use of firearms in U.S. Pacific Islands and among AANHPI populations. A key informant in Hawaii noted: “I think that if you look at the methods that are used for suicide among [AANHPI] populations, it very much does not fall in line with what you read in the national report.” This key informant noted that firearm injury is “easily the number one method [among Veterans overall]…but it’s significantly lower in [AANHPI] populations. Hanging is, suffocation is the top…it’s the number one in Hawaii data, the Hawaii data that we have on Veterans.” This key informant indicated that these differences likely extend to individuals residing in Guam, the Northern Mariana Islands, and American Samoa, noting that hanging reflected “80-something percent of all age suicide deaths in Guam…and the same thing I hear from people in Saipan as well and American Samoa.”

Indeed, a key informant in Guam discussed how the emphasis on firearm suicide prevention in the U.S. does not reflect the predominant suicide method used in Guam and among Pacific Islanders: “When you think about tailoring [LMS] to local spaces, we know that on Guam, people are dying by hanging, that’s the primary means. And so, if you look at national data or national strategies towards LMS, it’s focused on firearm safety…But it’s very different for Pacific Islanders, at least on Guam, and I think on the other islands. I’m not sure about Pacific Islanders in the mainland, but I would say locally, here on Guam, it is very seldom are people dying by gunfire.” These differences were discussed by another key informant in Guam, who noted a potential reason for these differences: “Lethal means definition in CONUS is handguns, right? On Guam, it’s rope. And maybe opiates, ice, whichever. But mostly hanging because, again, it being a negative, they don’t wanna draw attention. If you shoot yourself, it’s reported in the news. If it’s a hanging, no one knows except the family, the first responders, medical examiner, and the funeral home.”

Although mention of the increased use of hanging was only discussed by key informants in Hawaii and Guam, a few key informants in CONUS noted cultural differences in suicide methods in the broader AANHPI population. For example, a key informant in CONUS referenced prior research on the lower use of firearms as a suicide method among Asian Americans: “Asian Americans have lower rates of usage than White folks, and when they die by suicide, they’re also less likely to use firearms.”

#### “*That protective factor is slowly eroding”*: Lower rates of firearm access and concern about increasing rates of firearm ownership.

Key informants discussed lower firearm ownership in the Pacific Islands and in the AANHPI population as potential reasons for the decreased use of firearms as a suicide method in this region and population. However, they also noted that there have been recent increases in firearm ownership regionally and in subsets of the AANHPI population, expressing concerns about how this may impact suicide risk.

For example, noting that “hanging is above firearms” in AANHPI suicide deaths, a key informant in Hawaii indicated that this may be due to “limited firearms in Hawaii.” Another key informant in Hawaii noted regional and cultural differences in firearm purchasing and ownership: “That’s not a big thing in Hawaii, owning guns and using them…I guess people don’t buy guns here that much…in [city], I don’t remember dealing with guns too much with Asian Americans… that’s more prevalent with the Caucasian population.” However, another key informant in Hawaii noted that there has been an increase in local firearm ownership: “[The] island has really gone into the Second Amendment, everybody’s got guns now. They’ll talk about it. They get a lot of training on guns and safety and a lot of ‘em do the range.”

Other key informants pointed to broader, cultural explanations for lower rates of firearm ownership in the AANHPI population, such as laws regarding firearm ownership in different Asian countries. A key informant in CONUS stated:

“I think it’s just a function of the countries in which Asian Americans immigrate from, where there is much less of a gun culture…It’s just societal. You don’t have the Second Amendment right in these countries. So you have a lot more laws that restrict, severely restrict, the use of firearms. It’s like countries that nobody can own a gun unless you are serving in the military or serving within a police force. Gun usage is severely restricted. So you get immigrants coming from these countries, they don’t have the familiarity with guns…So you have that embedded in your culture. But that’s changing.”

Reiterating this, a key informant in Hawaii discussed how there are “strict gun laws and stuff that’s hard to get in Japan and Korea. You can’t buy [a gun] over there.” Nonetheless, a key informant in CONUS discussed how being an immigrant could also increase one’s sense of danger and desire to have a firearm for protection: “I think a lot of Asian American and Pacific Islander Veterans are typically a lot more conservative than people realize…I think people forget immigrants, lower income folks, like those are the people that sometimes feel unsafe…some people actually want guns to defend themselves ‘cause they feel like they live in a community where they need that safety.”

While key informants noted that firearm ownership has traditionally been lower in the AANHPI population, some noted that this appeared to be changing. For example, a key informant in CONUS indicated that firearm ownership increased among Asian American and Pacific Islanders during the COVID-19 pandemic due to experiences that led to fear and a desire for self-protection: “I read gun ownership among AAPI has increased by like 40% or something in the past year or so, and I think about how much of that is due to the Sinophobia, xenophobia, Asian hate that was just so prolific and widespread during the onset of COVID...to the point where like our elders were getting attacked everywhere and the fear that that breeds.” This key informant noted concern that this fear “results in desire to protect yourself, and then people buy guns to protect themselves, but then they’re not actually addressing the underlying mental health needs of our community, and we’re not actually addressing the conflict resolution and relationship skills and all these things that are required to like improve mental health more broadly.” This confluence – obtaining firearms without addressing the root causes of mental health concerns and community needs – was noted to potentially lead to perilous situations as “when these conflicts come up, as they inevitably do, they also have this increased access to deadly weapons.”

Another key informant in CONUS indicated that there has been increased interest in firearms among younger Asian Americans, particularly men, and suggested that this may result in increased suicides in this population over time: “Younger Asian Americans are becoming more interested, especially men, in using firearms. So it’s kind of like gun culture is becoming more prevalent.” They continued, “If that trend continues, you’re gonna find a rise in suicide among Asian Americans.” This key informant attributed the rise in firearm interest within this population to societal perceptions regarding masculinity, noting that “owning guns has been shown as one of the ways people project, men project their masculinity.”

Thus, while key informants’ explanations for the potential increases in firearm ownership among AANHPI populations varied, there was concern expressed about the implications of increased firearm access on suicide risk in this population. A key informant in CONUS stated: “One protective factor Asian Americans have – and that protective factor is slowly eroding – is a lack of access or conditional lack of access to guns and firearms…The greater access you have, the greater likelihood that you feel like you can use firearms.”

#### *“What resonates to us here and how do we use it?”*: Research and guidance needed to inform tailored, targeted LMS efforts.

Key informants acknowledged the need for research to inform LMS approaches to be responsive to AANHPI Veterans, including the contexts in which they reside. In response to a question about what research is most needed to prevent suicide among AANHPI Veterans, a key informant based out of CONUS noted the importance of research to understand what suicide methods AANHPI Veterans are using: “I think manner of suicide would be informative. Because I don’t know if guns are primarily used or not. There’s some interesting studies of others, not Veterans, but AAPI cultures. They have different ways of [dying by] suicide. And so, if you’re trying to reduce means for suicide, you would target those things versus guns or whatever. So I think those are the important questions.” Thus, identifying differences in suicide methods among AANHPI Veterans was seen as a vital step to suicide prevention for this population. Similarly, a key informant in Hawaii also discussed the need for research on suicide methods among AANHPI Veterans. “I’d like to see more research delineated to method types with this population so that we can understand it better. Even though we work with this every day to some degree or every week or month…we’re still trying to understand that...We’re trying to take what national data is saying. But then how does that translate or work locally?” This effort to translate national level data and strategic goals to local culture and needs was seen as paramount: “I’m trying to think, okay, how would I extrapolate that to local culture? Like what does that look like for us here when I look at that data?” They elaborated, while also noting the need for research to be disaggregated to reflect regional differences:

“I think getting the research certainly more specific to our islands and territories here, and not always necessarily having to lump in the research always with, like I don’t think it’s fair to put AANHPI, always lumping them in the same as AANHPI on the mainland. Because those Veterans are often very different if they’ve been born and raised in the mainland versus the ones that live here in the islands. It’s a different group.”

**Hanging.** A key informant in Guam described how they work with other providers in Guam to incorporate questions about hanging into suicide prevention:

“I’m reminding them about if this individual is having suicide ideations and they’re developing their Safety Plan with them, how can you remind them about—or ask the questions about access to low hanging rods like closets? How can you bring up the conversation of, ‘Is there someone available at home to keep—are we able to keep the doors open to every room in the house so that there’s a clear sightline amongst the rooms? Is there a storage shed anywhere around the house?’ We see people who are dying by suicide and those outdoor storages that so many people on Guam have and hanging themselves from a space there. ‘Are there strong, large trees on your land? What kind of rope is available?’ Just things like that.”

Another key informant in Guam noted the importance of ensuring that suicide deaths are documented as suicides in mortality surveillance data, indicating that this may be complicated when a suicide attempt does not immediately result in death: “If you die because of a complication from an attempted suicide, that’s not tracked…You attempted [suicide] by hanging on a Monday, you don’t pass away until a week later, it’s recorded by the medical examiner’s office as a kidney or liver failure, it’s not reported as a suicide.”

Key informants also noted the need for research on hanging. A key informant in Hawaii emphasized the need for research on interventions to prevent suicide by hanging, particularly considering the ubiquitous nature of ligatures and the challenges to preventing suicide by hanging: “I would like to see more emphasis being put upon how do we educate the public? How do we look at future interventions of what we could do? Because I know ligature is not an easy one to address because it’s so easy compared to, not everyone owns a firearm, right? But most people own a rope of some kind or an extension cord or something that could be used in that context. So that’s something that I would definitely like to see happen for these populations.”

**Firearms.** A key informant in CONUS stressed the need to understand rates of firearm ownership among AANHPI Veterans, stating: “That would be something I’d be intrigued in from a suicide perspective is gun ownership among Asian American, Pacific Islander Veterans because…statistically the horrible numbers that ‘guns plus suicide’ equals.” A CONUS key informant described how cultural considerations regarding firearms could be incorporated into prevention efforts:

“At a more primary or universal prevention level, even before you escalate to suicide ideation, I think that there should be campaigns and preventative activities…that encourage men, Asian American men, to think of masculinities in ways that are more flexible that go beyond guns…this is where you can use a more culturally relevant [perspective]…to say that we come from cultures where we’ve been able to resort to ways of addressing conflicts and disputes without guns. And that’s where—in many Asian countries, that’s how it’s done. So, kind of emphasize that component, that this is something that is, in a way, foreign to Asian American cultures. To be able to emphasize that at a primary prevention level and not just wait ‘til someone is actively suicidal.”

## Discussion

Considering the substantial increase in suicide rates among AANHPI Veterans (i.e., by 193% from 2001 to 2021 [2]), it is essential to understand how to prevent suicide through the methods most commonly used in suicide deaths among AANHPI Veterans. Although a recent quantitative study demonstrated that there are differences in suicide methods between AANHPI Veterans, compared to the overall Veteran population [6], to our knowledge, no prior studies have sought to understand this issue among clinicians who work with AANHPI Veterans and other subject matter experts who may have insights into suicide prevention with AANHPI Veterans. This secondary analysis of interview excerpts from subject matter experts in CONUS, Hawaii, and Guam provides initial insights into LMS needs among AANHPI Veterans. Although the interview guide did not include specific questions about LMS, the guide provided opportunities for LMS content to emerge, and approximately forty percent of key informants in this sample provided responses relevant to LMS among AANHPI Veterans. Key informants discussed differences in suicide methods between AANHPI Veterans and the overall Veteran population, as well as regionally, that may be important for tailoring LMS efforts. Specifically, efforts may benefit from a broader view of LMS, including a greater emphasis on the prevention of hanging, among AANHPI Veterans and Veterans residing in the Pacific Islands. Key informants also attributed lower firearm injury in the AANHPI population to historically lower rates of firearm ownership, yet were concerned that recent increases in firearm ownership in this population may heighten suicide rates, including rates of firearm suicide. Lastly, key informants indicated that research is needed to inform tailored suicide prevention for AANHPI Veterans, including by region.

### Hanging

Key informants noted the increased use of hanging as a suicide method among AANHPI populations and in the Pacific Islands. However, this was only mentioned by key informants in Guam and Hawaii, which may reflect the heightened use of hanging as a suicide method in these regions. Hanging accounted for 82% of suicide deaths in Guam from 2012–2022 [9], and suffocation (the broader category that includes hanging) accounted for 50.8% of suicide deaths in Hawaii in 2022 and was the most common suicide method [26]. For comparison, suffocation accounted for 24.2% of suicide deaths in the U.S. in 2022 and was the second most common suicide method [1]. Although suffocation is used more as a suicide method in Pacific Island regions [9,26], suffocation accounted for 38.5% of AANHPI Veteran suicide deaths in 2019, second only to firearm injuries [6]. Suffocation is also a highly lethal method [27] that is used across U.S. regions [7] and which is important to prevent. As no key informants in the CONUS subsample mentioned hanging or suffocation, this may suggest a need to understand—and potentially increase—the extent to which practitioners involved in AANHPI Veteran suicide prevention in CONUS are aware of the heightened use of suffocation as a suicide method in this population. Future research would help with better understanding this.

These findings also underscore the importance of developing suicide prevention trainings and resources specific to a wide array of lethal means, including hanging, which may be particularly relevant for (but not limited to) practitioners working with AANHPI Veterans in the Pacific Islands. Key informants highlighted an important challenge to developing and implementing interventions specific to ligatures—most notably, the ‘ubiquity’ of everyday items that could be used for hanging. Indeed, the widespread availability of such items, as well as their potential necessity and utility in daily life, presents a significant practical challenge for clinicians aiming to encourage at-risk Veterans to reduce their access to lethal methods of suicide. Additional work is needed to understand the types of ligatures used in suicide attempts and deaths, the varying lethality of each, and approaches that are impactful in limiting access to preferred ligatures among at-risk Veterans which do not lead them to attempt suicide by other highly lethal methods (e.g., firearms, other ligatures).

### Firearms

Firearm injury is the most common method of suicide death among AANHPI Veterans (i.e., accounted for 40.8% of suicide deaths in 2019 [6]) and was the most common suicide method discussed by key informants. However, firearm injuries account for a lower percentage of suicide deaths among AANHPI Veterans, relative to among Veterans overall [6]. AANHPI individuals in the general U.S. population also had the lowest rates of firearm suicide from 2019–2022, relative to other racial and ethnic groups [28]. Some key informants appeared to be cognizant of these differences, noting the lower use of firearms as a suicide method among AANHPI populations and in specific regions (e.g., Hawaii, Guam), which they attributed to historically lower rates of firearm ownership. It was suggested that lower firearm ownership among Asian Americans may reflect some individuals having immigrated to the U.S. from countries where firearm ownership is less common. Indeed, the rate of firearm suicide is lower among AANHPI individuals born outside the U.S., compared to those born in the U.S. [16], with research showing that individuals born outside the U.S., specifically from countries with a low-to-medium rate of firearm possession, are less likely to use firearms as a suicide method [29]. Research examining this among AANHPI Veterans, however, has yet to occur.

Key informants also expressed concerns that the suicide rate may increase in the AANHPI population due to recent increases in firearm ownership. They noted concern about increased firearm ownership in specific subsets of the AANHPI population, including among younger Asian American men and elderly Asian American individuals. Indeed, rates of firearm suicide increased from 2018–2021 among AANHPI youth [30]. Additionally, among AANHPI Veterans, firearm injury accounted for higher percentages of suicide deaths from 2005–2019 among those who were in the youngest (ages 18–34: 52.5%) and oldest (≥75: 69.5%) age groups, relative to among those in the middle age groups (35–54: 43.4%; 55–74: 43.9%) [6]. Research also supports the presence of sex differences in the use of firearms as a suicide method, both in the overall Veteran population [1] and among AANHPI Veterans specifically [6], with firearm injury accounting for a higher percentage of suicide deaths among males than females. As increases in household firearm ownership temporally precede increases in firearm suicide rates [31], examining how firearm ownership trends have changed over time in the AANHPI Veteran population—and by age and sex—is warranted.

Regarding recent trends in firearm ownership among AANHPI populations, key informants discussed recent events, such as racism during the COVID-19 pandemic, as a potential explanation for increased firearm ownership among AANHPI populations, a finding supported by extant research. A national 2020 survey found that individuals who reported experiencing racism during the COVID-19 pandemic were 2.6 times as likely to report purchasing a firearm, compared to those who did not experience racism during the pandemic [32]. Additionally, in a 2020–2021 survey, Asian Americans reported an increase in both first-time firearm ownership and carrying a firearm since the pandemic began; those who experienced racial discrimination were more likely to purchase a firearm and ammunition, and those who experienced more anticipatory racism-related stress indicated elevated intent to purchase firearms [33]. As firearm research specific to AANHPI Veterans remains sparse, epidemiological research to examine factors impacting firearm access, as well as to better understand trends in firearm ownership among AANHPI Veterans over time, is warranted.

### Clinical implications and future research

Our findings underscore the importance of continuing to attend to cultural and regional differences when considering AANHPI Veterans’ suicide prevention needs. Only a few key informants described how they incorporate differences regarding suicide methods into their approach to LMS (such as by asking questions regarding ligatures and ligature points). Understanding the most effective ways to approach LMS with AANHPI Veterans is an important next step for future research. Extant research has identified specific differences that may be important to consider in LMS approaches with AANHPI Veterans, particularly considering the wide-ranging perspectives that individuals may hold regarding potential lethal means of suicide [34]. Additionally, AANHPI individuals are more likely to use handguns, but less likely to use other firearm types (e.g., rifles, shotguns), in intentional self-harm [35]. A broader analysis of interviews with subject matter experts suggested that it is important to consider the roles of shame, stigma, and culturally-relevant idioms of distress in conceptualizing suicide risk and prevention with AANHPI Veterans, while underscoring the importance of implementing suicide prevention in settings where AANHPI Veterans access care (e.g., primary care) [5]. Interviewing AANHPI Veterans about their perspectives regarding LMS approaches is a critical next step to understanding their preferences for LMS discussions (e.g., who to have these discussions with and in which settings), as well as to elucidate the specific beliefs and contextual factors driving use of specific suicide methods. Beliefs about hanging and its impact on one’s body and on others may be one factor that contributes to individuals attempting suicide through hanging [36]; nonetheless, as these beliefs may vary in different populations and regions, understanding if these beliefs extend to AANHPI Veterans and if there are unique cultural aspects to such beliefs, may help with developing resources to prevent suicide by hanging in this population.

### Limitations

Given the broader focus of interviews on subject matter experts’ perspectives, experiences, and recommendations regarding AANHPI Veteran suicide prevention, the semi-structured interview guide was designed to elicit information deemed relevant to suicide prevention broadly, with no questions explicitly included about LMS. While this approach did elicit responses relevant to LMS (including in response to multiple different questions), it is possible that the lack of specific questions about suicide methods or LMS in the interview guide resulted in a reduced number of responses regarding such topics and that explicit inclusion of such questions would have elicited additional, richer information. Future research designed *a priori* to focus specifically on LMS with AANHPI Veterans is warranted. It is also important to acknowledge that some interviews were conducted prior to information on methods of suicide among AANHPI Veterans being published [6,7,10]. Another limitation is that, while we interviewed key informants from various regions where AANHPI Veterans reside, we did not interview any key informants from American Samoa, the Northern Mariana Islands, or Compacts of Free Association nations, which have substantial AANHPI Veteran populations [17,37]. Future research that specifically focuses on LMS for AANHPI Veterans across a variety of regions is warranted.

## Conclusions

These findings are among the first to discuss subject matter experts’ perspectives regarding LMS considerations among AANHPI Veterans, which add important context and depth to prior epidemiological findings on differences in suicide methods among AANHPI Veterans [6] and how these differ by region [7]. Results provide insights on differences in LMS needs in this understudied, historically underserved population, particularly surrounding the prevention of hanging, as well as the potential for increasing firearm suicide deaths over time among AANHPI Veterans. Findings also yield information regarding potential gaps in knowledge and areas for future research, such as increasing awareness among practitioners in CONUS that hanging is a more common method of suicide death among AANHPI Veterans, increasing resources to prevent suicide by hanging, and understanding if rates of firearm ownership and firearm suicide are indeed changing in this population. Development and pilot testing of tailored LMS approaches to prevent suicide among AANHPI Veterans are warranted.

## Supporting information

S1 Appendix Qualitative Interview Guide. (PDF)
