# Supplementary material for: Lethal means safety for Asian American, Native Hawaiian, and Pacific Islander Veterans: Insights from key informants
Source: PLoS One. 2026 Jan 30;21(1):e0340962. doi: 10.1371/journal.pone.0340962 (PMC12857927; doi:10.1371/journal.pone.0340962)
Supplement: S1 Appendix — (PDF) [file pone.0340962.s001.pdf]

<u>Qualitative Interview Guide</u>

As is conventional for qualitative research using semi-structured interviews for data collection, the exact wording of questions and probes varied. The questions and probes listed are representative of the general content explored in each interview. However, not every question was necessarily asked of every participant depending on interview flow and timing. ***Content in brackets was modified, depending on the relevance to the individual being interviewed. Interviewers were instructed to probe throughout and modify wording based on whether the participant was providing expertise regarding Asian American (AA) Veterans, Native Hawaiian (NH), Pacific Islander (PI) Veterans, or any combination thereof.]***

1. **How did you find out about this research study? What motivated you to participate?**

2. **Please describe your current job/role.**

3. **Please tell me about your experiences working with AANHPI <u>individuals</u> (regardless of whether they have been Veterans).** *[Clarify focus of this interview regarding the subsequent questions]*

4. **What comes to mind when you think of:**
   a. … AANHPI populations you have worked with?
   b. …AANHPI Veterans?

5. **Please tell me about your experiences working with AANHPI Veterans.** *[Skip if their experience has not included Veterans]*
   a. When did you begin working with AANHPI Veterans? What was that like for you?
   b. Do you work with AANHPI Veterans in your current position? If so, what does this entail?
   c. Can you tell me more about the specific AANHPI populations you have worked with? What has that been like for you?
   d. In what types of settings have you worked with AANHPI Veterans? In which regions have these Veterans resided? Have your experiences working with AANHPI Veterans varied based on the geographical location or region you were in? If so, please describe.
   e. [For interviewees who are <u>researchers</u>] Please tell me about research you have conducted related to AANHPI Veterans.
   f. [For interviewees who are <u>clinicians</u>] Please tell me about the clinical care you have provided to AANHPI Veterans. What percentage of your patients are AA or PI Veterans?
      i. What trainings or resources have helped you address the specific needs of these groups?

6. **Do you think that AANHPI Veterans' experiences before, during, and after their military service differ, compared to Veterans of other racial or ethnic backgrounds? If so, please describe.**
   a. Are there any experiences that are unique to specific AA or PI Veteran communities? For example:
      i. How, if at all, do experiences of Asian American Veterans differ from those of Pacific Islander Veterans?
      ii. How about experiences among specific Asian American Veteran communities?
      iii. What about experiences among specific Pacific Islander Veteran communities?

7. **What cultural norms or values are important for understanding suicide risk and prevention with AANHPI Veterans?**
   a. Are there specific cultural norms or values that are important to consider amongst AANHPI Veterans?
   b. How well do current suicide prevention interventions and resources incorporate these?
   c. To your knowledge, are any current suicide prevention interventions and resources inconsistent with these?

8. **To what extent is suicide discussed among AANHPI Veterans? If it is discussed, how is it discussed? Are there any important considerations for how to discuss suicide risk and prevention with AANHPI Veterans, including with respect to the specific words or language used?**

9. **We are interested in understanding more about suicide, including risk and protective factors, among AANHPI Veterans, including any that may be unique to (or particularly important for) AANHPI Veterans.** *[Probe throughout: How might these be similar or different to non-AANHPI Veterans?]*
    a. In your experience, what factors cause AANHPI Veterans to experience suicidal thoughts and behaviors? Which factors cause AANHPI Veterans to be at risk for suicide? What factors do you think are important to consider in understanding why some AANHPI Veterans die by suicide?
    b. What factors <u>protect</u> against suicide among AANHPI Veterans?
    c. How do you think risk and protective factors for suicide differ between Asian American compared to Pacific Islander Veterans? Are there differences that are important for clinicians and researchers to consider?
    d. Similarly, are there important differences within specific Asian American or Pacific Islander groups with respect to suicide risk and protective factors that should be considered?

10. **To your knowledge, what is currently being done to prevent suicide among AANHPI Veterans?** [Optional follow-up probe: what is being done to address the risk factors you mentioned earlier?]
    a. How effective are these initiatives for preventing suicide among AANHPI Veterans?
    b. What aspects are helpful?
    c. What aspects are not helpful?
    d. What else would help?
    e. To what extent do you think that existing suicide prevention interventions and resources for AANHPI Veterans address their needs, values, and cultures? Can you think of ways that such interventions or materials have been adapted?

11. **What else do you think needs to be done to prevent suicide among AANHPI Veterans?**
    a. <u>Where</u> should suicide prevention interventions for AANHPI Veterans be delivered to be most effective? (For example, within VA healthcare settings, community settings, or elsewhere)
    b. <u>Who</u> should deliver such interventions?
    c. What would help with implementing these interventions? What trainings or materials would help?
    d. What could get in the way of providing these interventions?

12. **Are there any important <u>geographical</u> considerations for preventing suicide among AANHPI Veterans <u>in your region</u>? For example, barriers to providing suicide prevention interventions or materials in your region? Facilitators?**

13. **[For healthcare providers only] Have you conducted a <u>Safety Plan</u> (sometimes known as a Crisis Response Plan) with AANHPI Veterans?** *(If yes, ask the questions below)*
    a. What was that like for you?
    b. Did your experience conducting Safety Plans with AANHPI Veterans differ in any way from conducting Safety Plans with Veterans of other racial/ethnic backgrounds?
    c. What did you find to be <u>helpful</u> when conducting Safety Plans with AANHPI Veterans?
    d. What did you find to be <u>unhelpful</u> or <u>challenging</u> when conducting Safety Plans with AANHPI Veterans?
    e. Did you ever receive any guidance or training on conducting Safety Plans with AANHPI Veterans specifically?

f. Are there any considerations specific to conducting Safety Plans with AANHPI Veterans <u>in your region</u> that would be important for us to know about?
g. What would you recommend or suggest to other healthcare providers who conduct Safety Plans with AANHPI Veterans?
h. What questions do you have related to conducting Safety Plans with your AANHPI Veteran patients? What would support you in doing this?

14. Are you familiar with <u>Caring Contacts</u>? *(If yes, ask the questions below)*
a. Have you ever used Caring Contacts or any elements of it with AANHPI Veterans? If so, what was that like for you? What did you find to be <u>helpful</u> when using Caring Contacts with AANHPI Veterans? What did you find to be <u>unhelpful</u> or <u>challenging</u> when using Caring Contacts with AANHPI Veterans?
b. Did you ever receive any guidance or training on using Caring Contacts with AANHPI Veterans specifically?
c. What considerations do you think would be important for using Caring Contacts with AANHPI Veterans? Barriers? Facilitators?
d. Are there any considerations specific to using Caring Contacts with AANHPI Veterans <u>in your region</u> that would be important for us to know about?
e. Who should deliver the Caring Contact messages when working with AANHPI Veterans in your region?
f. What would you recommend or suggest to other healthcare providers who use Caring Conducts with AANHPI Veterans?
g. What questions do you have related to using Caring Contacts with your AANHPI Veteran patients? What would support you in doing this?

15. How, if at all, should mental health and suicide prevention clinical care differ when working with AANHPI Veterans?
a. What things do you think providers should be aware of or account for?
b. What trainings, if any, do you think could be beneficial for those working with AANHPI Veterans?
c. What factors do you think are important to account for during mental health therapy or counseling (e.g., evidence-based psychotherapy, Safety Planning) and suicide risk assessment and prevention with AANHPI Veterans?

16. What resources do you think AANHPI Veterans would find most appropriate/useful/worthwhile for suicide prevention?
a. What resources do you find helpful when working with AANHPI Veterans?
b. What resources do you wish existed?
c. In what formats should these be offered?
d. Who should offer them?
e. Where should they be offered?

17. What <u>research</u> is most needed to prevent suicide among AANHPI Veterans?
a. In your opinion, what is the single most important research question to investigate to help prevent suicide among AANHPI Veterans?
b. What challenges are important to consider in addressing this research question?
c. What would help in overcoming these challenges?

18. **Is there anything else you think is important to share?**
